# Downregulated miR-29a/b/c during Contact Inhibition Stage Promote 3T3-L1 Adipogenesis by Targeting DNMT3A

**DOI:** 10.1371/journal.pone.0170636

**Published:** 2017-01-23

**Authors:** Yingjie Zhu, Guangyong Zheng, Huichao Wang, Yudong Jia, Ying Zhang, Yanfeng Tang, Wenlong Li, Yanan Fan, Xiaodong Zhang, Youwen Liu, Sanhong Liu

**Affiliations:** 1 Henan Provincial Luoyang Orthopedic-Traumatological Hospital (Henan Provincial Orthopedic Hospital), Luoyang, Henan, China; 2 CAS-MPG Partner Institute for Computational Biology, Chinese Academy of Sciences, Shanghai, China; 3 Shanghai Institute for Advanced Immunochemical Studies, ShanghaiTech University, Shanghai, China; Tohoku University, JAPAN

## Abstract

Differentiation of 3T3-L1 cells into adipocytes involves a highly-orchestrated series of events including contact inhibition (CI), clonal expansion, growth arrest, and terminal differentiation. Recent study demonstrated that 3T3-L1 preadipocytes will not be differentiated into mature adipocytes without CI stage, which indicated that CI stage plays an important role during 3T3-L1 adipogenesis. However, the molecular mechanism is not yet fully understood. In the present study, we found that the expression level of miR-29a/b/c was decreased and the expression of DNMT3A was up-regulated during CI stage, respectively. Furthermore, overexpression of miR-29a/b/c during CI stage inhibits adipogenesis significantly but not at other stages. In addition, miR-29a/b/c repressed DNMT3A expression by directly targeting its 3’ untranslated region (3’ UTR). Our data reveal a novel mechanism of miR-29a/b/c in the regulation of adipogenesis.

## Introduction

The prevalence of overweight and obesity in developed and developing countries has greatly increased the risk of insulin resistance and type 2 diabetes mellitus. Growth arrest, clonal expansion and terminal differentiation of preadipocytes are required for generation of mature adipocytes [1]. These processes are controlled by a complex network of transcription factors, including peroxisome proliferator-activated receptor γ (PPARγ), CCAT/enhancer binding proteins, Krupple-like factors and sterol regulatory element-binding proteins, as well as extracellular hormones [2, 3]. Guo et al. showed that adipocytes were barely detected from the population of the cycling 3T3-L1 cells without contact inhibition under inducing conditions [4], which indicated that CI stage is prerequisite for adipocyte differentiation. However, the mechanisms coordinating this step remains unknow.

MiRNAs are single-stranded, non-coding RNAs, ~21–23 nucleotides in length, which posttranscriptionally regulate the expression of multiple target genes [5]. Inhibition of enzymes involved in miRNA biogenesis, such as Drosha and Dicer, repressed the differentiation of human mesenchymal stem cells into adipocytes [6], which supports a role for miRNAs in adipocyte development. A major function of miRNAs in adipose tissue is to stimulate or inhibit the differentiation of adipocytes, and to regulate specific metabolic and endocrine functions [7, 8]. The functions of miRNAs as stimulators or inhibitors of murine and/or human adipocyte differentiation programmes have been reviewed in detail elsewhere [9–15]. For example, let-7 was the first human miRNA discovered [16], which has been reported in adipogenesis, with overexpression of let-7 in pre-adipocytes resulting in reduced adipogenesis by targeting HMGA2, thereby regulating the transition from clonal expansion to terminal differentiation [17, 18]. MiR-143 was first identified as a positive regulator of human adipocyte differentiation in 2004 by regulating ERKS signaling [19] and miR-143 is the only miRNA to date shown to be similarly regulated during human and mouse adipocyte differentiation [20]. Li et al. reported that miR-17-5p was increased during human adipose-derived mesenchymal stem cell adipogenesis *in vitro* and that miR-17-5p mimic transfection resulted in enhanced adipogenesis in the same cell population by repressing bone morphogenetic protein 2 (BMP2) and increased CCAAT/enhancer-binding protein alpha (C/EBPα) and peroxisome proliferator-activated receptor gamma (PPARγ) expression [21]. miR-miR-27 is a negative regulator of adipocyte differentiation via suppressing PPARγ [22, 23] and cAMP responsive element binding protein (CREB) expression [24]. TNFα-induced up-regulation of miR-155 inhibits adipogenesis by down-regulating early adipogenic transcription factors CCAAT/enhancer-binding protein beta (C/EBPβ) and CREB [25]. In addition, the roles of miR-21 [26], miR-22 [27], miR-130 [28], miR-221/222 [29], miR-200 [30], and miR-223 [31] have been investigated in many studies. Although so many miRNAs were found to regulate the differentiation of adipocytes, it is still not clear whether miRNAs are involved in the process of contact inhibition to regulate the differentiation of adipocytes.

In the present study, we found that the expression of miR-29a/b/c is down-regulated during CI stage in 3T3-L1 cells, and overexpression of miR-29a/b/c, especially during CI stage, inhibited 3T3-L1 differentiation. Simultaneously, the expression of DNA methyltransferase DNMT3A (de novo methyltransferases) was elevated during CI stage. In addition, miR-29a/b/c regulated DNMT3A expression by binding its 3’ UTR in directly manner. These results demonstrated that miR-29a/b/c can be a potential target for the treatment of obesity.

## Materials and Methods

### Cell culture and differentiation

Mouse embryonic fibroblast-derived 3T3-L1 preadipocytes were maintained and differentiated in a 37°C incubator with 10% CO_2_ as previously described [24, 25]. Differentiated 3T3-L1 adipocyte monolayers were analyzed with an Oil-Red-O staining assay.

### Western blotting

The protocol was used as previously described [4]. All primary antibodies were incubated with the membrane at 4°C overnight: DNMT3A (Santa Cruz Biotechnology, sc-20703), and tubulin (Sigma, T6199). Membranes were washed with 1×TBST and incubated with either anti-mouse or anti-rabbit IgG horseradish peroxidase-conjugated secondary antibody (Santa Cruz Biotechnology).

### RNA isolation and real-time PCR analysis

Total RNA of 3T3-L1 cells was isolated by using miRVana Isolation Kit according to the manufacturer’s instructions (Ambion). 0.5 μg total RNA from each sample was reverse-transcribed into cDNA using the PrimeScript^™^ RT reagent Kit (Takara). The miRNA levels were quantitatively assessed by SYBR Green-based quantitative real-time PCR with gene-specific primers in an Applied Biosystems PRISM 7900HT Fast Real-Time PCR System according to the manufacturer’s instructions (Applied Biosystems). U6 was used as an internal normalization control.

### Luciferase reporter transfection and dual luciferase assay

In the 3’ UTR-reporter assay, 293T cells were grown to 80%-90% density in 24-well plates and were then transfected with 50 ng of the 3’ UTR reporter (pGL3-DNMT3A), 20 ng of the transfection control Renilla vector (pRL-TK, Promega), and 100 nM miRNA precursor molecules (Ambion) along with 1 μl of Lipofectamine 2000. Lysates were harvested 48 hours after transfection, and reporter activity was measured with the Dual Luciferase Assay (Promega). Relative luciferase levels were calculated with the formula (S_luc_/S_renilla_)/(C_luc_/C_renilla_), where Luc is raw firefly luciferase activity, renilla is the internal transfection control of renilla activity, S is the sample, and C is the control pre-scramble.

### Microarray

For mRNA microarray, total RNA of 3T3-L1 cells was isolated with an RNeasy Total RNA Isolation kit (QIAGEN). The overall gene-expression profiles were detected by Affymetrix Mouse 430 2.0 according to Affymetrix GeneChip expression-assay protocols. For miRNA microarray, total RNA of 3T3-L1 cells was isolated by using miRVana Isolation Kit according to the manufacturer’s instructions (Ambion). The miRNA expression profiles were detected by Affymetrix miRNA 2.0 Array. Three independent experiments and two independent experiments were done for mRNA and miRNA (We originally had three biological repeats on miRNA array, but one of the sample had problems, so we only selected two biological repeats for further analysis), respectively.

## Results

### Contact inhibition is required for 3T3-L1 differentiation

**Fig 1:**
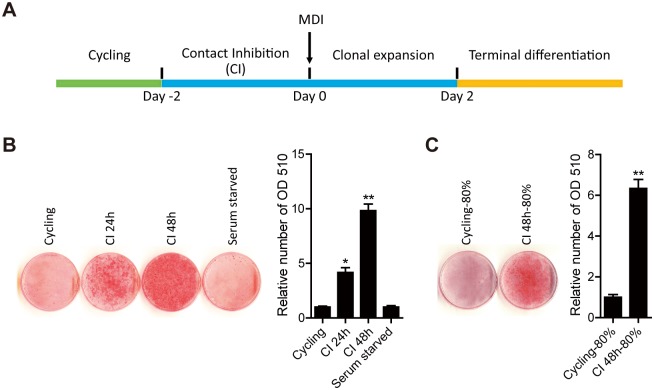
Contact inhibition is required for the differentiation of preadipocytes. (A) Schematic for the process of 3T3-L1 cell differentiation. (B) Cycling, CI 24h, CI 48h and serum starved preadipocytes were subjected to adipogenesis induction. On day 8 of the induction, differentiated cells were stained with Oil Red O to measure the levels of triglyceride droplets. (C) CI 48h preadipocytes were re-cultured at 50% cell density then grew to 80% and cycling preadipocytes at 80% cell density were subjected to adipogenesis inducing conditions. The Oil Red O staining cells were extracted with isopropanol and measured at OD 510.

As mentioned in previous studies, 3T3-L1 differentiation includes several stages: cycling, contact inhibition, clonal expansion and terminal differentiation (Fig 1A). To determine the role of the contact inhibition stage for adipocyte differentiation, we induced the 3T3-L1 cell at cycling, CI 24h, CI 48h stages. The results demonstrated that more 3T3-L1 cells were differentiated into adipocytes when they stayed in CI stage longer, whereas adipocytes were rarely detected from the cycling 3T3-L1 cells without contact inhibition under the same inducing conditions (Fig 1B). In order to determine whether the adipocyte differentiation is induced by cell cycle arrest, we also analyzed the differentiation of 3T3-L1 cells with serum starved conditions, the results showed that adipocytes were not detected in serum starved conditions (Fig 1B). In addition, the 3T3-L1 cells after 48h contact inhibition were reseeded at 50% density then grew to 80% density were subjected to the differentiation induction condition, adipocyte differentiation was observed in these reseeded 3T3-L1 cells. However, the cycling 3T3-L1 cell reseeded at 80% density were not differentiated into adipocytes (Fig 1C). Taken together, these results demonstrated that contact inhibition stage is required for the differentiation of 3T3-L1 cells.

### The expression profiles of mRNA and miRNA during CI stage

**Fig 2:**
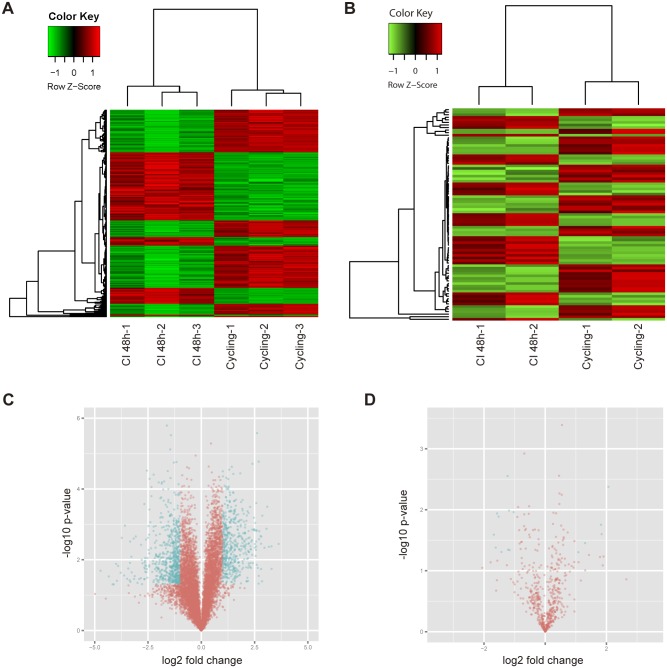
Differentially expressed genes and miRNAs in CI 48h and cycling preadipocytes. (A) Gene-cluster analysis of genes differentially expressed in CI 48h and cycling preadipocytes. Each row represents a single gene. Red, genes with higher expression levels; green, genes with lower expression levels. *p* < 0.05. (B) MiRNA-cluster analysis of miRNAs differentially expressed in CI 48h and cycling preadipocytes. Each row represents a single miRNA. *p* < 0.05. (C) The volcano plot analysis for the mRNA microarray data of CI 0h and CI 48h. (D) The volcano plot analysis for the miRNA microarray data of CI 0h and CI 48h.

In order to understand the molecular mechanism of 3T3-L1 cells during the contact inhibition stage, we detected the expression profiles of mRNA and microRNA from CI 0h to CI 48h. For mRNA, expression ratio from CI 0h to CI 48h was adopted as criteria to identify different expression genes. In practice, 278 up-regulated genes and 334 down-regulated genes were identified with a threshold of ratio fold change more than 2.0 and p-value less than 0.05 (Fig 2A, 2C and S1 Table). While for miRNA, a similar criterion based on expression ratio was utilized to pick out significant change miRNAs. As a result, 46 up-regulated miRNAs and 37 down-regulated miRNAs were picked out with a threshold of ratio fold change more than 1.5 or p-value less than 0.05 (Fig 2B, 2D and S2 Table). Given the abundance of miRNAs in 3T3-L1 cells, three down-regulated miRNAs: miR-29a, miR-29b and miR-29c were selected for further study.

### The signaling pathway associated with CI stage

**Fig 3:**
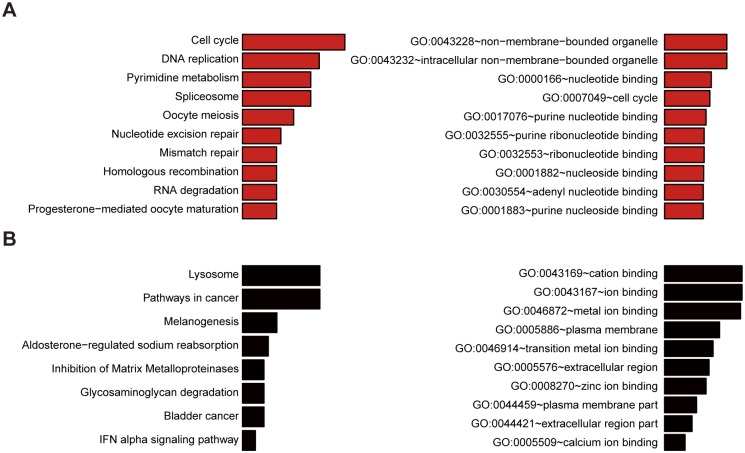
The pathway analysis of up- or down-regulated genes during CI stage in 3T3-L1 cells. (A) KEGG pathway analysis (left) and gene ontology analysis (right) of down-regulated genes during CI stage. (B) KEGG pathway analysis (left) and gene ontology analysis (right) of up-regulated genes during CI stage.

In order to understand the signal pathway associated with the contact inhibition stage, different expression genes identified from mRNA microarray data were selected for the pathway analysis. The results showed that cell cycle is the most obvious in the signaling pathway involved in the down-regulation of genes (Fig 3A). It is easy to understand this result because the 3T3-L1 cell cycle will be blocked during CI stage. Lysosome pathway is the most significant pathway related to up-regulation of genes (Fig 3B). As for the GO analysis, many nucleotide binding were involved in the down-regulation of genes (Fig 3A), whereas many ion binding were involved in the up-regulation of genes (Fig 3B), which indicated that nucleotide binding and ion binding are crucial for CI stage.

### The expression level of 29a/b/c was decreased and DNMT3A was increased during CI stage

**Fig 4:**
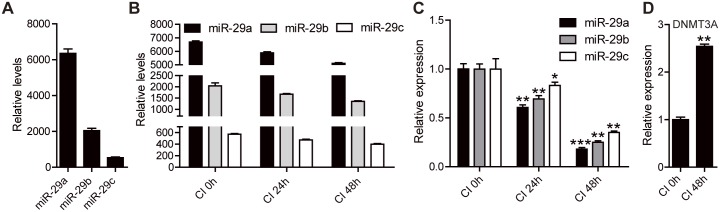
The expression levels of 29a/b/c and DNMT3A during CI stage. (A) The expression abundance of miR-29a, miR-29b and miR-29c in CI 48h preadipocytes. (B) The expression abundance of miR-29a, miR-29b and miR-29c in CI 0h, CI 24h and CI 48h preadipocytes. (C) The real-time PCR results of miR-29a, miR-29b and miR-29c in CI 24h and CI 48h preadipocytes compared with CI 0h. * *p* < 0.05, ** *p* < 0.01, *** *p* < 0.001. (D) The expression level of DNMT3A mRNA in CI 48h compared with CI 0h. ** *p* < 0.01.

Among the all down-regulated miRNAs during CI stage, we found that the abundance of miR-29a/b/c are very high in 3T3-L1 cells (Fig 4A), which indicated these three miRNAs might play important roles in 3T3-L1 cells. As shown in Fig 4B, the abundance of miR-29a/b/c was decreased during CI stage, the same results were confirmed by real-time PCR (Fig 4C). In addition, the abundance of miR-29a is higher than miR-29b/c, and the reduction of miR-29a is more significant than miR-29b/c, which implies that miR-29a is more important than miR-29b/c during CI stage (Fig 4A–4C). Among the up-regulated genes, DNMT3A was significantly elevated during CI stage (Fig 4D). The previous studies also showed that DNMT3A plays an important role in the adipocyte differentiation [4].

### Overexpression of miR-29a/b/c in CI stage inhibits 3T3-L1 cells differentiation

**Fig 5:**
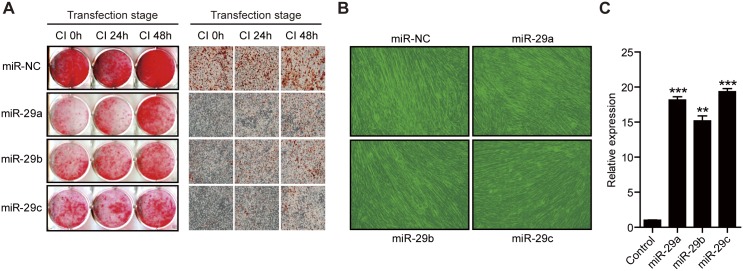
Overexpression of miR-29a/b/c inhibits 3T3-L1 cells differentiation. (A) Preadipocytes were transfected with 100 nM miR-NC, miR-29a, miR-29b or miR-29c for the indicated time periods and then subjected to adipogenesis induction. (B) C2C12 myoblast cells were transfected with the indicated miRNAs. Myogenic differentiation was initiated at 48 h post-transfection by maintaining the cells in culture medium containing 2% horse serum. (C) Detection of exogenous miR-29a, miR-29b and miR-29c expression in C2C12 myoblast cells. 100 nM miR-NC, miR-29a, miR-29b or miR-29c was transfected in C2C12 for 48 h then their expression were assessed by an SYBR green-based quantitative real-time PCR. ** *p* < 0.01, *** *p* < 0.001.

Given the expression of miR-29a/b/c was decreased during CI stage, we transfected miR-29a/b/c mimics into 3T3-L1 cells at different stages: CI 0h, CI 24h and CI 48h (Fig 5A) to detect which stage is the most important stage for miR-29a/b/c. The results showed that 3T3-L1 differentiation was inhibited significantly when miR-29a/b/c mimics were transfected in CI 0h or CI 24h stage (Fig 5A). This results indicated that miR-29a/b/c play a more important role in CI stage but not at other stages. Furthermore, the inhibitory effect of miR-29a on adipocyte differentiation was significantly better than that of miR-29b/c (Fig 5A).

To further determine whether miR-29a/b/c inhibits adipogenesis specifically, we investigated the effect of miR-29a/b/c on the myogenic differentiation of the C2C12 myoblast cells. Formation of myofibers was not significantly affected by miR-29a/b/c overexpression (Fig 5B and 5C), indicating that miR-29a/b/c does not play an important role in myogenic differentiation.

### miR-29a/b/c inhibits 3T3-L1 differentiation by directly targeting DNMT3A 3’ UTR

**Fig 6:**
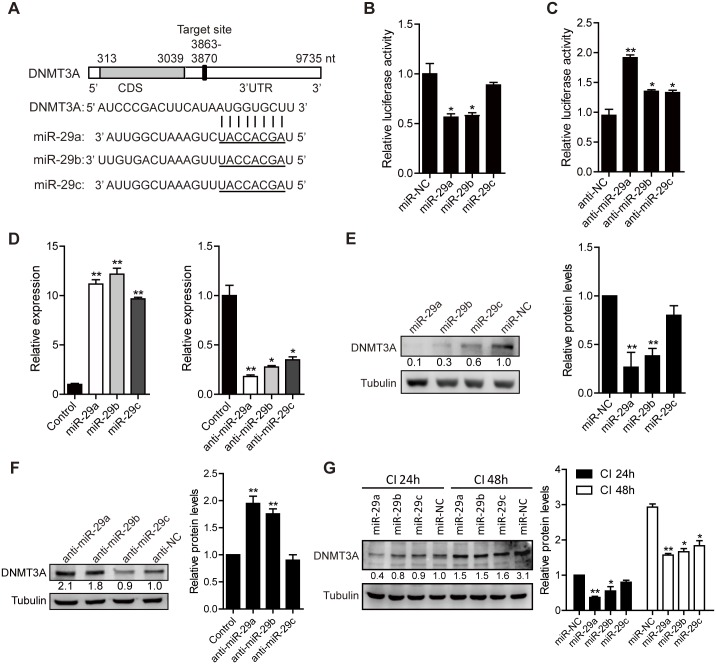
Overexpression of miR-29a/b/c suppresses DNMT3A expression in directly manner. (A) Schematic diagram of predicted miR-29a/b/c target site in the 3’ UTR of DNMT3A. (B) MiR-29a/b/c regulate DNMT3A by directly binding to its 3’ UTR. 293T cells in 24-well plates were transfected with 3’ UTR reporter (50 ng), renilla vector (pRL-TK, 20 ng), and miRNA mimics (100 nM). After 24 h transfection, the cells were collected for dual-luciferase assay. (C) Anti-miR-29a/b/c elevates the luciferase activity of pGL3-DNMT3A. 3T3-L1 cells in 24-well plates were transfected with 3’ UTR reporter (50 ng), renilla vector (pRL-TK, 20 ng), and miRNA antisense (100 nM). After 48 h transfection, the cells were collected for dual-luciferase assay. * *p* < 0.05, ** *p* < 0.01. (D) The overexpression or inhibition efficiency of miR-29a/b/c mimics (left) or antisense (right) in 3T3-L1 cells. 100 nM miR-29a/b/c mimics or antisense was transfected in 3T3-L1 cells, the expression of miR-29a/b/c was detected by real-time PCR after 48 h transfection. * *p* < 0.05, ** *p* < 0.01. (E)—(F) MiR-29a/b/c regulate DNMT3A protein level. 80% density 3T3-L1 cells were transfected with 100 nM miR-NC, miR-29a, miR-29b, miR-29c mimics (E) or antisense (F) for 48 h. The expression of DNMT3A was analyzed by western blotting. Tubulin was used as a loading control. The data were presented as means ± SEM. ** *p* < 0.01. (G) 3T3-L1 cells were transfected with 100 nM miR-NC, miR-29a, miR-29b or miR-29c mimics in CI 0h stage, the cells were collected in CI 24h or CI 48h stage for western blotting analysis. The data were presented as means ± SEM. * *p* < 0.05, ** *p* < 0.01.

DNMT3A play a critical role in CI stage of 3T3-L1 differentiation [4]. By searching miRNA target prediction websites TargetScan, miRanda and PicTar, DNMT3A 3’ URT contains a miR-29a/b/c binding site that is partially complementary to miR-29a/b/c (Fig 6A). We performed a luciferase reporter assay to test whether miR-29a/b/c directly binds DNMT3A 3’ UTR. The results demonstrated that miR-29a/b overexpression significantly inhibited luciferase activity of pGL3-DNMT3A, whereas miR-29c overexpression did not affect the luciferase activity significantly (Fig 6B). In addition, inhibition of miR-29a/b/c increased the luciferase of pGL3-DNMT3A (Fig 6C).

We also detected the transfect efficiency of miR-29a/b/c precursor molecules or miR-29a/b/c antisense, the results showed that the expression of mature miR-29a/b/c increased 9–12 fold or decreased 2.5–5 fold relative to the control (Fig 6D). DNMT3A is partially down-regulated or up-regulated in the transfected miR-29a/b/c precursor or antisense 3T3-L1 cells (Fig 6E and 6F). Furthermore, we transfected miR-29a/b/c precursor in CI 0h, then detected the expression level of DNMT3A in CI 24h and CI 48h, the results showed that DNMT3A expression was repressed with overexpression of miR-29a/b/c (Fig 6G). Simultaneously, the protein level of DNMT3A in CI 48h is higher than CI 24h, which is consistent with the mRNA level during CI stage (Figs 6G and 4F).

## Discussion

The obesity epidemic has focused attention on adipose tissue and the development of fat cells. Adipogenesis, or the development of fat cells from preadipocytes, has been one of the most intensely studied models of cellular differentiation [2]. There are several stages are required for generation of mature adipocytes including growth arrest, clonal expansion and terminal differentiation of preadipocytes [1, 7]. These processes are regulated by a complex network of transcription factors [2, 3]. Guo et al. found that CI stage was essential for 3T3-L1 cells differentiation. The differentiation licensing of 3T3-L1 cells during CI stage was mainly involved in epigenetic modifications such as DNA methylation. They showed that DNMT3A (a key enzyme involved in DNA methylation) siRNA transfection during the contact-inhibition stage significantly reduced adipogenesis efficiency [4]. We also found that the expression of DNMT3A was up-regulated during CI stage. However, the molecular mechanism of regulating DNMT3A still remains to be clarified.

In our previous study, we demonstrated that miR-155 and miR-27 play a role in the early stage of adipocyte differentiation by suppressing C/EBPβ or CREB expression [24, 25]. Whether miRNAs regulate 3T3-L1 cells differentiation in an earlier stage, such as CI stage, which is still unclear. In the present study, the miRNAs expression profiles were examined by miRNA microarray analysis of 3T3-L1 cells from CI 0h and CI 48h. We identified 46 up-regulated miRNAs, and 37 miRNAs that were down-regulated relative to CI 0h. Among them, the miR-29 family (29a, 29b, and 29c) with the high abundance was down-regulated significantly. MiR-29 family has intriguing complementarities to the 3'-UTRs of DNMT3A [32]. Our results demonstrated that miR-29 regulated DNMT3A expression by binding its 3’ UTR in directly manner in 3T3-L1 cells.

Since 2007, miR-29 has been known to negatively regulate insulin signaling in adipocytes [33], however, the role of miR-29 in 3T3-L1 cells differentiation is still not clear. We overexpressed miR-29a/b/c in different stages (CI 0h, CI 24h, CI 48h) of 3T3-L1 differentiation, it is obvious that over expression of miR-29a/b/c during the whole CI stage can significantly inhibit the differentiation of 3T3-L1 cells (Fig 5A). In addition, the effect of miR-29a on the differentiation of 3T3-L1 cells was more significant than that of miR-29b/c (Fig 5A), consistent with the regulation of miR-29a and miR-29b/c on DNMT3A expression (Fig 6). These results indicates that even if these miRNAs belong to the same family, there is still a difference in the regulation of the same gene.

Taken together, our results provide a new perspective into the role of miR-29 in the differentiation of 3T3-L1 cells. *In vivo* investigation will be necessary to evaluate the precise role of miR-29 in the inhibition of adipocyte differentiation.

## Supporting Information

S1 Table The different expression genes during contact inhibition stage in 3T3-L1 cells. (XLSX)

S2 Table The different expression miRNAs during contact inhibition stage in 3T3-L1 cells. (XLSX)
